# A Novel Intensity-Based Approach to Increasing Prefrontal Cerebral Oxygenation by Walking Exercise

**DOI:** 10.3390/jpm12040510

**Published:** 2022-03-22

**Authors:** Ya-Wen Hsiao, Hsin-Ya Tzeng, Chi-Ming Chu, Hsiang-Yun Lan, Hui-Hsun Chiang

**Affiliations:** 1 School of Nursing, National Defense Medical Center, No 161, Sec. 6, Minquan E. Road, Neihu Dist., Taipei 11490, Taiwan; AAAA0190702@gmail.com (Y.-W.H.); a0920366201@gmail.com (H.-Y.T.); shinnylan@gmail.com (H.-Y.L.); 2 Department of Nursing, Tri-Service General Hospital, No 325, Sec. 2, Chenggong Rd., Neihu Dist., Taipei 10490, Taiwan; 3 Division of Biostatistics and Medical Informatics, Department of Epidemiology, School of Public Health, National Defense Medical Center, Taipei 114201, Taiwan; chuchiming@web.de; 4 Graduate Institute of Life Sciences, National Defense Medical Center, Taipei 114201, Taiwan; 5 Graduate Institute of Medical Sciences, National Defense Medical Center, Taipei 114201, Taiwan; 6 Big Data Research Center, Fu-Jen Catholic University, New Taipei City 242062, Taiwan; 7 Department of Public Health, School of Public Health, China Medical University, Taichung 404328, Taiwan; 8 Department of Public Health, Kaohsiung Medical University, Kaohsiung 807378, Taiwan

**Keywords:** cardiac force meter, prefrontal cerebral oxygenation, hemoencephalography, regional cerebral blood flow, walking exercise

## Abstract

Regional cerebral blood flow (rCBF) increases after moderately intense exercise and is significantly correlated with cognitive function. However, no intensity-based physiological indicator for enhancing rCBF during low- to-moderate-intensity exercise has been proposed. The purpose of this study was to develop a physiological indicator housed in a wearable device to determine whether low-to-moderate intensity walking can increase rCBF. A cross-sectional study with four parallel arms was performed. Each of 114 participants was randomly assigned to either the moderate, low-to-moderate, low, or very low walking intensity groups. A novel dynamic cardiac force meter (CFM) was used to quantify walking intensity. Heart rate and hemoencephalography (HEG) were measured during each phase of the session. Compared to baseline, HEG significantly increased in both the submaximal exercise and recovery phases in members of the low-to-moderate intensity group but not the very low intensity group. Low-to-moderate intensity walking improves prefrontal cerebral blood oxygenation. The present results demonstrate the usefulness of a dynamic CFM housed in a wearable device for quantifying the intensity of walking exercise aimed at increasing prefrontal blood oxygenation. The results of the study may help guide further development of exercise strategies for brain disease patients and the ageing population.

## 1. Introduction

The WHO 2020 guidelines on physical activity and sedentary behavior reaffirm that reducing sedentary behavior, and even low intensity exercise, is beneficial for all age groups [1]. The guidelines indicate that physical activity is considered especially beneficial for older people and those living with disabilities, such as those suffering from stroke or traumatic brain injury, and also may increase regional cerebral blood flow (rCBF) and improve cognitive function [2,3]. Moderately intense exercise is strongly encouraged in older adults with cognitive impairment or mobility issues [4,5]. A meta-analysis showed that increased physical activity has a small but significant effect in improving cardiometabolic risk factors [6]. In a three-group randomized controlled trial, 67 sedentary obese older adults who engaged in a morning session of moderately intense exercise were found to have significantly improved executive function compared to a control group that engaged in prolonged sitting, whereas a group that engaged in low-intensity walking interspersed with sitting breaks showed significant improvement in working memory, but not executive function compared to the control group [7].

rCBF has been proposed as a prognostic indicator for cognitive and neuropsychiatric symptoms [8]. Regional CBF, used as an index of prefrontal regional brain oxygenation, was found to increase during moderate intensity exercise in healthy adults, but decrease during high intensity exercise, which can lead to a deficit in cognitive function [9,10]. Greater prefrontal CBF has been associated with better performance on executive function and attention tasks, whereas poor cerebral hemodynamics have been associated with cognitive decline [11]. rCBF alternation has been associated with cognitive dysfunction as well as subsequent cognitive recovery in patients with traumatic brain injury [12], Alzheimer’s disease [13], and mild cognitive impairment [14]. Cerebral hypoperfusion has been associated with risk of dementia, and lower baseline cerebral perfusion has been associated with accelerated decline in cognition [15]. CBF was less and cognitive performance was poorer in healthy older adults than in healthy youngsters. A significant positive relationship was found between rCBF and cognitive performance when age was controlled [16].

Much evidence exists that exercise improves rCBF both during and after exercise in people of all ages or people with such disabilities as vascular brain injury, especially if the exercise is moderately intense. Twelve weeks of moderately intense aerobic exercise were found to alter rCBF in older adults with mild cognitive impairment and improve their performance on cognitive tasks [17]. Moderately intense aerobic exercise was found to regulate rCBF in chronic stroke patients [18]. rCBF was found to increase during a 20-min session of moderately intense aerobic exercise, and regional perfusion in the hippocampus was found to increase up to 65 min after this exercise in healthy participants [19]. Intense exercise increases rCBF and cerebral oxygenation; the latter effect increases with age because of a reduction in rCBF and is greater in men than in women [20,21]. A recent study suggests that aerobic fitness is associated with lower CBF and greater cerebrovascular reactivity in healthy young adults than in older adults [22]. Little is known about the relevance of rCBF in healthy young adults and whether the effects of moderate to low intensity exercise are different in healthy young adults than in older adults.

These studies suggest that moderately intense exercise is important for improving cognitive function because it increases rCBF. Advances in near-infrared spectroscopy (NIRS) and hemoencephalography (HEG) enabled the use of a dynamic measure of cerebral blood oxygenation as well as other homodynamic measures to monitor changes in regional cerebral blood oxygenation [23]. HEG is a useful tool for assessing prefrontal-dependent functions in a clinical context. It measures blood flow within the brain and is used to monitor and train cerebral function by recording the HEG (prefrontal CBF) data without the inconvenience of electrodes [24]. Although the HEG device cannot be applied anywhere except to the forehead scalp and cannot measure temporal effects [24], our use of it has demonstrated that the increase in average brain blood oxygenation is positively correlated with improvement in brain function and other long-lasting physiological changes [25]. Moreover, wearable devices can be used to improve the quality of physical activity by providing feedback of physiological measurements and performing contact tracing more efficiently than non-wearable devices, especially in the era of COVID-19 [26]. Wearables have been shown to be highly feasible and effective tools in the service of physical fitness [27]. They improve fitness tracking with optical sensors because the heartbeats are represented as pulse waves.

Although the available devices differ in how accurately they measure exercise intensity, wearable fitness trackers are a useful way for people to assess their physical activity [28]. However, there are currently no wearable intensity-based dynamic physiological indicators that measure increases in rCBF during low to moderately intense physical exercise, which is easy for people to engage in. We aimed to fill this knowledge gap by developing such a device.

## 2. Materials and Methods

### 2.1. Participants

To avoid the influence of age, gender, and health status on normative hemodynamic changes, healthy young adults in a narrow age range were randomly assigned to four exercise intensity groups with equal numbers of males and females in each group. Persons were eligible for the study if they were healthy, 20 to 25 years old, and able to provide informed consent. Persons were excluded if they had any known cardiovascular or neuromuscular disease, were taking any prescription medication, or had a BMI in the obese range (>30 kg/m^2^). It was estimated that a total of 80 participants for the four groups would be required to obtain a clinically meaningful within-participants interaction between exercise condition and data-collection time on a measure of cerebral oxygenation; specific requirements for an adequate sample size for the study were Cohen’s *f* of 0.48 [29], power of 0.8, and alpha of 0.05. To allow for a 33% measurement-failure rate, we recruited 120 potential participants, 60 males and 60 females, through advertisements at a university in Taipei, Taiwan. As 6 of these were excluded because of poor availability of physiological measures for people who are underweight, the final sample was 114 participants.

### 2.2. Study Design

We employed a cross-sectional design with four parallel arms. Thirty of the 120 participants were randomly assigned to each of four groups defined by how vigorously they were asked to walk during the exercise phase of the study: “moderate intensity”, “low-to-moderate intensity”, “low intensity”, and “very low intensity”. To account for the confounding influence of sex, we employed an Internet-based randomization program (www.thesealedenvelop.com, accessed on 18 January 2021) to stratify assignments to the exercise intensity groups by sex in a uniform 1:1 ratio with a block size of 4.

### 2.3. Procedure

At the beginning of the session, all participants filled out a questionnaire asking about sex, age, marital status, patterns of regular exercise, number of exercise sessions per week, duration of each exercise, and habits of smoking and drinking. A period of 15–20 min was assigned for participants to complete the questionnaire and to establish a steady state before the session. Sleep quality was assessed by asking: “How would you evaluate your sleep quality in the last one month?” The response alternatives ranged from 1 (“very bad”) to 4 (“very good”). All participants were informed that they would be asked to walk on a treadmill for 30 min with heart rate monitoring and cerebral blood oxygenation measurement.

The rest of the session was divided into five phases: baseline resting (5 min), warmup walking (5 min), submaximal walking exercise (20 min), cool down (5 min), and recovery (10 min). All the phases were conducted in a soundproof room with temperature 24–28 °C and during 8:00 a.m. to 8:00 p.m. time of day to control for circadian variability. During all phases, prefrontal cerebral oxygenation and heart rate were measured as described below.

At the end of the 5-min baseline period, during which the participants sat in a quiet and comfortable room, their blood pressure was measured by a sphygmomanometer that had been clinically calibrated according to the European Society of Hypertension International Protocol [30]. At the end of the exercise phase, blood pressure was again taken.

The participants were walking in some fashion during the warmup, exercise, and cool down (30 min total). All this walking was performed on a treadmill (T7000 Pro; Johnson, Taipei, Taiwan). Heart rate readings from a fitness tracker housed in a wearable heart rate monitor were recorded every second. The intensity of the walking exercise, operationally defined by heart rate, was monitored for each participant by the cardiac force meter of the fitness tracker. The readings of the fitness tracker and the chest-band wearable device were confirmed to be equivalent in the warmup phase. Walking in the warmup and cool down phases was leisurely. During the exercise, participants were asked to walk at the level of intensity corresponding to the condition they were assigned to before the session. At the end of the exercise phase, participants gave a rating of perceived exertion (RPE) using the Borg scale, which has a response range of 6 to 20 for submaximal walking [31]. After the 20-min aerobic exercise phase and the 5-min cool down phase, there was a 10-min recovery period during which participants were asked just to sit in a quiet and comfortable room.

### 2.4. Measures

#### 2.4.1. Heart Rate

Heart rate during all the phases was monitored by a chest-band-wearable device (BioHarness 3, Zephyr Technology, Annapolis, MD, USA) which monitors and records posture and activity level in addition to heart rate, heart rate reserve (HRR: maximum heart rate during the exercise minus the resting heart rate), respiration rate, and estimated core temperature [32]. It functions as a wireless ambulator accelerometer that records dynamic heart response as electrical impulses (beats per minute) and sends them, along with the other measures, by Bluetooth to an electronic register also on the device in real time [33]. The Zephyr Bioharness has been approved for clinical use by the US Food and Drug Administration [34], and a systematic review confirms that the device provides reliable and valid measurements of heart rate in multiple contexts [35].

#### 2.4.2. Fitness Tracker

A wearable fitness tracker (Garmin vivosmart 4, Garmin, Taipei, Taiwan) was used to monitor the intensity of participants’ walking on a treadmill during the exercise period. The tracker’s cardiac force meter (CFM) provided the physiological feedback participants needed to attain and maintain the assigned exercise intensity level for 20 min.

#### 2.4.3. Prefrontal Cerebral Oxygenation

HEG ratios were obtained through a biofeedback software system (Thought Technology INFINITY, BioExplorer, BioEra, Brain Master, Matlab) that measures oxygen blood flow to the prefrontal cortex, using near-infrared spectroscopy [36]. Thus, HEG provides continuous, noninvasive, and quantitative assessment of cerebral hemodynamics and oxygenation, the values of which have been shown to be positively correlated with those from single photon emission computed tomography [23].

#### 2.4.4. Exercise Intensity

Intensity of activity during the entire 30-min session was defined and quantified which is derived from a measure of centimeters traveled per heartbeat (CMPB) by standardizing the centimeter measure to 3 km per heartbeat.

$$\mathrm{CMPB}=\frac{\nu_{\mathrm{cm}/\min}}{\mathrm{heart}\;\mathrm{rate}};\;\mathrm{CFM}=\mathrm{CMPB}\times \frac{3\;\mathrm{km}}{\nu_{\mathrm{Km}/\mathrm{hr}}}$$

These measures are based on a cardiac force index for estimating the dynamic cardiac function developed and reported by Hsiao et al. [37]. The lower the value of cardiac force meter (CFM), the greater the exercise intensity. A CFM value of 35 represents “jogging” intensity, 40 represents “race walking” intensity, 45 represents “breeze walking” intensity, and 50 represents “leisure walking” intensity.

For the purposes of analysis, group membership was defined not by the walking instructions the participant was given before the session but by the participant’s CFM value for the exercise during the session: (1) moderate intensity, <37.5; (2) low-to-moderate intensity, 37.5–42.5; (3) low intensity, 42.6–47.5; (4) very low intensity, >47.5. This is because we considered CFM to be a better representation of the energy expended during, and thus the intensity of, the exercise than the instructions given to the participants beforehand. The instructions had been to attain adequately variability across the entire sample in the actual intensity of the exercise.

### 2.5. Statistical Analyses

Results on the participant characteristics and clinical measures were summarized as frequencies, percentages, means, and standard deviations. Multiple linear regression using a forward stepwise method was performed to examine the effects of the participant characteristics on baseline HEG. Analysis of variance and chi-square tests were carried out to examine the homogeneity of these characteristics across the groups. As adequate homogeneity was demonstrated, changes in the outcome measures from baseline to recovery, and group differences in these changes, were tested using the generalized estimating equation (GEE). The alpha criterion for significance was set at *p* < 0.05, two-tailed.

## 3. Results

### 3.1. Participant Characteristics

Data on participant demographics and habitual behavior are presented in Table 1. Half of the participants were male (*n* = 59, 51.8%) and the overall mean age was 22.56 years (*SE* = 0.14). None of the participants acknowledged drinking or smoking. The most frequent numbers of days a week of exercise were two (*n* = 35, 30.7%) and three (*n* = 27, 23.7%). Most participants reported that their exercise sessions lasted 31–60 min (*n* = 43, 37.7%). About one fifth (*n* = 21, 18.4%) admitted to not exercising regularly. As for the intensity of exercise in the experimental session, most participants (*n* = 43, 37.7%) fell in the low group, with CFM between 42.6 and 47.5, and the fewest participants (*n* = 19, 16.7%) fell in the very low group, CFM > 47.5.

### 3.2. Intensity of Walking during the Session

In the original group classification, 29 (25.9%), 27 (24.1%), 28 (25%), and 28 (25%) participants fell in the moderate, low-to-moderate, low, and very low group, respectively. In the new classification, four moderate group members entered the low-to-moderate group, six low-to-moderate group members entered the low intensity group, and nine very low intensity group members entered the low intensity group. The majority of participants (*n* = 43, 37.7%) fell in the low intensity group, with CFM between 42.6 and 47.5, and the fewest participants (*n* = 19, 17.0%) fell in the very low intensity group, CFM > 47.5. The other two participants in each group (*n* = 26, 22.8%) fell in the moderate and low-to-moderate intensity group.

**Figure 1:**
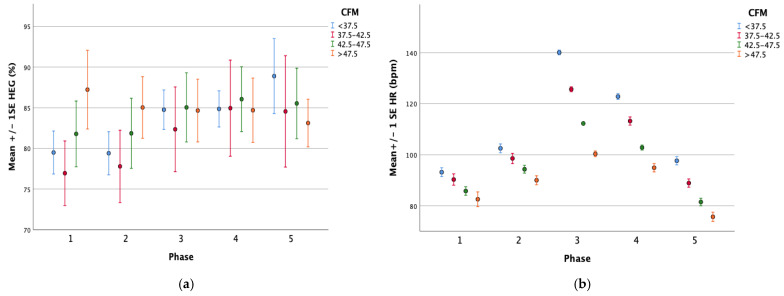
Changes in HEG (**a**) and HR (**b**) across session phases in the four groups. CFM, cardiac force meter; HR, heart rate; HEG, hemoencephalograph. Phase 1, baseline; Phase 2, warm up; Phase 3, submaximal walking exercise; Phase 4, cool down; Phase 5, recovery. (**a**) Changes in HEG between session phases. (**b**) Changes in HR between session phases.

As shown in Figure 1, for all four groups both HEG and heart rate increased from the preceding phase in all the phases except for the cool down phase and the recovery phase, in which HEG increased but heart rate decreased.

As shown in Table 2, for the moderate intensity group (CFM < 37.5) mean HEG was 79.49% in the baseline phase and increased to 84.75% in the exercise phase, whereas mean heart rate increased from 93.2 bpm in baseline to 140.1 bpm in exercise. However, mean HEG increased from 84.75% in exercise to 84.85% in cool down and further to 88.89% in recovery, while mean heart rate decreased from 140.1 bpm in exercise to 122.8 bpm in cool down and further to 97.6 bpm in recovery.

To the contrary, in the very low intensity group (CFM > 47.5), mean HEG was 87.23% in baseline and then decreased to 84.66% in exercise, whereas mean heart rate increased from 82.6 bpm in baseline to 100.3 bpm in exercise. However, HEG decreased from 84.66% in exercise to 83.12% in recovery, while heart rate decreased markedly from 100.3 bpm in exercise to 75.7 bpm in recovery.

We also analyzed the intensity of exercise in terms of HRR and RPE. The means and standard errors of HEG and heart rate for each of the four groups for each phase of the session are presented in Table 2. The mean HRR percentages across all phases for the very low, low, low-to-moderate, and moderate groups were 20.96%, 29.20%, 40.40%, and 49.20%, respectively.

### 3.3. Associations between Baseline HEG and Personal Habits

We further examined how baseline HEG differed as a function of personal habits. As shown in Table 3, the multiple linear regression analysis revealed a significant independent positive association between number of days a week of exercise and baseline HEG across the four groups (β = 0.22, *p* < 0.05). There was a significant main effect of weekly frequency of exercise on baseline HEG for the four groups combined, F = 4.30, *p* = 0.04.

### 3.4. Changes in HEG across Phases

As shown in Table 4, the GEE analysis revealed that across all groups HEG was significantly higher in recovery (*M* = 85.67, *SE* = 2.52) than in baseline (*M* = 81.07, *SE* = 2.04), χ^2^ = 6.64, *p* = 0.01. Moreover, the difference in mean HEG between recovery and baseline was significantly greater for the very low intensity group than for the moderate intensity group, χ^2^ = 5.79, *p* = 0.016. Planned comparison analyses revealed that the HEG values improved significantly for the moderate intensity group from baseline to recovery (*M*_base_ = 79.49 ± 13.46, *M*_rec_ = 88.89 ± 23.48; *t* = 3.18, *p* = 0.004), but the trend was opposite for the very low intensity group (*M*_base_ = 87.23 ± 21.10, *M*_rec_ = 83.12 ± 12.80; *t* = −0.92, *p* = 0.37).

A significant influence of weekly exercise on HEG was noted, χ^2^ = 6.90, *p* = 0.009. The difference in mean HEG between baseline and exercise was greater for the very low intensity group than for the moderate intensity group, but the difference was not significant after controlling for weekly frequency of exercise, χ^2^ = 3.55, *p* = 0.059. Planned comparison analyses revealed that HEG improved significantly from baseline to exercise for the moderate intensity group (*M*_base_ = 79.49 ± 13.46, *M*_exer_ = 84.75 ± 12.39; *t* = 6.10, *p* < 0.001) but non significantly worsened for the very low intensity group (*M*_base_ = 87.23 ± 21.10, *M*_exer_ = 84.66 ± 16.83; *t* = −0.61, *p* = 0.55).

In the supplement, we present the changes in HEG across phases separately for males (Table S1) and females (Table S2). In males, the change in mean HEG from baseline to recovery was significantly greater for the very low intensity group than for the moderate intensity group, χ^2^ = 5.26, *p* = 0.02, and there was a significant influence of weekly exercise on HEG, χ^2^ = 4.12, *p* = 0.04. In females, however, there was no significant difference in mean HEG from baseline to recovery for the very low intensity group compared to the moderate intensity group, χ^2^ = 1.26, *p* = 0.26, and there was no significant influence of weekly exercise on HEG, χ^2^ = 0.75, *p* = 0.39.

## 4. Discussion

Our results confirm the different effects of different intensities of exercise on increases in prefrontal cerebral oxygenation in healthy young adults. These results are consistent with those of previous studies we reviewed [18,38]. Our results are consistent with guidelines from the American College of Sports Medicine (ACSM) in showing that moderate, low-to-moderate-, low, and very low intensity levels of exercise were associated with heart rate reserve (HRR) ranges of 40–59%, 31–40%, and ≤30% respectively [39]. We observed a significant exercise intensity by session phase interaction effect on prefrontal cerebral oxygenation, as measured by HEG. Specifically, in the very low intensity group there was a significant reduction in prefrontal cerebral oxygenation from the walking phase to the recovery phase (which is the reverse of the other three groups), and a significant prefrontal cerebral oxygenation reduction from the warmup phase to the exercise phase but not from the exercise phase to the recovery phase.

We also demonstrated the usefulness of a recently developed cardiac force index indicator that uses CFM to quantify the intensity of exercise. The cardiac force index is based on the four levels of exercise intensity (moderate, low-to-moderate, low, and very-low) operationally defined by CFM. Monitoring cardiac status during exercise by calculating cardiac force index (CFI; weight * activity/heart rate) is the standard way to determine the workload of the heart during exercise [37]. The CFM, which is positively correlated with the reciprocal of heart rate, measures the workload of the heart and is the standard way to determine the intensity of exercise, which in turn determines the isometric level of the HRR. The real-time value of CFM can be used to quantify the exercise intensity level of one who is engaged in low or moderately intense exercise while wearing a device that reliably increases prefrontal cerebral oxygenation. Our results indicating that the standardized CFM can be used to divide exercise intensity into four categories (moderate, low-to-moderate, low, and very-low-intensity) and the ratio of HRR to heart rate during a walking exercise are consistent with those of previous studies [39,40].

Another of our findings suggests that a high frequency of exercise sessions is essential for increasing prefrontal oxygenation. Frequent exercise, even if low intensity, and a reduction in sedentary behavior are recommended by the global physical activity guidelines [1]. Although not reviewed in the Introduction section, results from previous research are consistent with this finding. Multiple exercise sessions per week have been shown to have significant benefits for cognitive performance [41]. A systematic literature review revealed that aerobically-trained healthy people attained high levels of cerebral oxygenation during intense exercise [29]. Recent studies indicate that even low intensity activity, if engaged in frequently, can improve glucose metabolism [42], cardiometabolic health [43] and cardiovascular health [44], as well as reduce mortality risk [45].

Moreover, our finding that prefrontal cerebral oxygenation increased for more than 10 min after a 20-min session of moderately intense aerobic exercise in healthy participants is consistent with results from a previous study [18]. An increase in regional cerebral uptake of oxygen accompanied by an increase in lactate taken up by the brain has been reported to occur during high intensity exercise. Brain glucose and O_2_ uptake were elevated, and lactate uptake remained high in the initial minutes of recovery immediately following the exercise [46]. These findings explain why cerebral blood oxygenation increased in the recovery phase of our study.

However, we found sex differences in the effects of both intensity of exercise and number of exercise sessions per week on increases in prefrontal cerebral oxygenation in healthy young adults. A recent study found that exercise increased cardiovascular reactivity in a sample of young adults [22]. Menstrual cycle and hormone levels may influence basal cerebral blood flow in young female adults [47]. These results suggest sex hormone effects as a cause of sex differences in prefrontal cerebral oxygenation changes.

### Limitations of Our Study

Several limitations should be noted in this study. To control the confounding factors of aging and health status, we limited our sample to young adults; further studies should examine the impacts of intensity-based exercise programs on the elderly or people living with a disability. Longitudinal evaluation of impacts from intensity-based exercise, rather than one-session evaluation, should be employed in further studies. Although methods of self-tracking intensity of physical activity for training purposes have improved considerably with the advent of heart rate measuring from the wrist, it is still necessary to establish the validity of these wearable devices. In particular, further research should investigate why different devices give different physiological indicators of CFM. Although near-infrared spectroscopy is a promising inexpensive and noninvasive alternative to single photon emission computer tomography (SPECT) or magnetic resonance imaging (MRI) for measuring prefrontal cerebral oxygenation, it still cannot provide information on absolute changes of blood flow in different regions of the prefrontal cortex. Although we adjusted for many confounders at baseline, several potential interference factors such as lifestyle, dietary intake, and environmental factors were not measured and thus not controlled for. Finally, health outcomes such as changes in cognitive function, mood, and quality of life should be measured in further studies to increase the robustness of the prefrontal cerebral oxygenation improvement norms.

## 5. Conclusions

In this paper we have presented norms for our four-level intensity-based prefrontal oxygenation enhancement protocol obtained from a physiological indicator of CFM housed in a wearable device. We used the device to demonstrate that low or moderate intensity walking exercises, but not very low intensity exercises, are effective in improving prefrontal oxygenation. Low or moderate intensity walking has added benefits, such as improving cognitive function mediated by the increased prefrontal oxygenation. Frequent engagement in low or moderate intensity exercise is an important way to improve prefrontal oxygenation in the general population. Our results may help guide further exercise strategies for the elderly or patients with brain disease.

## Figures and Tables

**Table 1:** Participant characteristics and group differences (*N* = 114).

			Exercise Intensity								*p* ^#^
			Moderate		Low/Moderate		Low		Very Low		
CFM:			<37.5		37.5–42.5		42.6–47.5		>47.5		
Variable	*n*	% (*M)* *	*n*	% (*M*) *	*n*	% (*M*) *	*n*	% (*M*) *	*n*	% (*M*) *	
Sex											
Male	59	51.8	14	53.8	13	50.0	22	51.2	10	52.6	0.99
Female	55	48.2	12	46.2	13	50.0	21	48.8	9	47.4	
Age *	22.56	0.14	22.33	0.29	22.63	0.26	22.62	0.23	22.55	0.14	0.86
BMI *	21.95	0.21	21.87	0.43	21.74	0.41	22.39	0.37	21.37	0.38	0.35
SBP *	115.45	1.14	114.1	1.91	117.0	2.65	115.3	1.88	115.6	3.09	0.87
DBP *	69.81	0.70	70.8	1.53	69.5	1.38	69.2	1.03	70.3	2.16	0.85
HR *	77.17	1.12	82.5	2.65	75.9	2.22	76.1	1.79	74.2	2.15	0.07
O_2_sat *	98.10	0.10	97.9	.21	97.8	0.20	98.2	0.14	98.6	0.24	0.05
Smoking											
No	114	100	26	100	26	100	43	100	19	100	
Yes	0	0	0	0	0	0	0	0	0	0	
Drinking											
No	114	100	26	100	26	100	43	100	19	100	
Yes	0	0	0	0	0	0	0	0	0	0	
Regular exercise											0.13
No	21	18.4	3	11.5	2	7.7	11	25.6	5	26.3	
Yes	93	81.6	23	88.5	24	92.3	32	74.4	14	73.7	
Sessions per week											0.32
1	13	11.4	5	19.2	4	15.4	4	9.3	0	0.0	
2	35	30.7	10	38.5	9	34.6	12	27.9	4	21.1	
3	30	26.3	5	19.2	8	30.8	10	23.3	7	36.8	
4	10	8.8	1	3.8	1	3.8	6	14.0	2	10.5	
5	5	4.4	2	7.7	2	7.7	0	0.0	1	5.3	
variable	21	18.4	3	11.5	2	7.7	11	25.6	5	26.3	
Duration of session (min)											0.32
<15	0	0	0	0	0	0	0	0	0	0	
15–30	28	24.6	10	38.5	8	30.7	6	13.9	4	21.1	
31–60	43	37.7	6	23.1	10	38.4	18	42.0	9	47.4	
>60	12	10.5	4	15.2	1	3.8	6	13.9	1	5.3	
variable	31	27.2	6	23.1	7	26.9	13	30.2	5	26.3	
Sleep quality											0.58
very good	16	14.0	5	19.2	3	11.5	5	11.6	3	15.8	
good	75	65.8	17	65.4	16	61.5	28	65.1	14	73.7	
bad	21	18.4	4	15.4	7	26.9	9	20.9	1	5.3	
very bad	2	1.8	0	0	0	0	1	2.3	1	5.3	

CFM, cardiac force meter; BMI, body mass index; SBP, systolic blood pressure; DBP, diastolic blood pressure, HR, heart rate, O_2_sat, oxygen saturation. * The values in the “%” columns are means for this variable. ^#^ From ANOVA or chi-square.

**Table 2:** Heart rate and prefrontal HEG changes between phases in the four groups (*N* = 114).

CFM: Measure	Phase	Exercise Intensity							
		Moderate		Low/Moderate		Low		Very Low	
		<37.5		37.5–42.5		42.6–47.5		>47.5	
		*M*	*SE*	*M*	*SE*	*M*	*SE*	*M*	*SE*
RPE		11.1	0.33	10.58	0.27	9.81	0.28	9.21	0.39
HRR (%)	Exercise	49.20	1.77	40.40	1.51	29.20	1.13	20.96	1.26
HR	Baseline	93.2	1.76	90.3	2.23	85.8	1.68	82.6	2.89
	Warmup	102.5	1.72	98.6	2.02	94.3	1.52	90.0	1.76
	Exercise	140.1	0.95	125.7	0.94	112.3	0.52	100.3	1.11
	Cool down	122.8	1.13	113.2	1.61	102.8	0.97	94.9	1.62
	Recovery	97.6	1.60	88.9	1.62	81.5	1.46	75.7	1.83
HEG	Baseline	79.49	2.64	76.95	3.97	81.78	4.04	87.23	4.84
	Warmup	79.41	2.66	77.79	4.45	81.85	4.31	85.03	3.29
	Exercise	84.75	2.43	82.34	5.21	85.04	4.25	84.66	3.86
	Cool down	84.85	2.22	84.95	5.91	86.06	3.98	84.69	3.96
	Recovery	88.89	4.61	84.55	6.85	85.53	4.34	83.12	2.94

CFM, cardiac force meter; HRR, heart rate reserve; HR, heart rate; HEG, hemoencephalograph; RPE, ratings of perceived exertion (RPE).

**Table 3:** Multiple regression of participant characteristics on baseline HEG.

Model	Total	Exercise Intensity (*M*/*SE*)				B	*SE*	*β*	*t*	*p*
		Mod	Low/Mod	Low	V Low					
Weekly frequency	2.56/0.11	2.35/0.24	2.50/0.23	2.56/0.17	3.00/0.23	4.51	2.18	0.22	2.07	0.04
Constant						70.23	6.03		11.64	<0.001

Age, sex, BMI, regular exercise or not, duration of exercise session, weekly frequency of exercise, sleep quality, and clinical measures (systolic blood pressure, diastolic blood pressure, heart rate, and oxygen saturation) at baseline and intensity group were entered using the forward stepwise method to select the independent variables for the model, *R*^2^ = 0.05. B, unstandardized coefficient, *β*, standardized coefficient.

**Table 4:** Mean changes in HEG from phase to phase of exercise session.

Variables	*β*	*SE*	χ^2^	*p*
Intensity * ^^^				
Moderate	Reference			
Low/Mod	−3.44	6.46	0.28	0.59
Low	2.95	5.78	0.26	0.61
Very Low	7.21	5.49	1.06	0.30
Phase ^#^				
Baseline	Reference			
Warmup	−0.09	1.95	0.002	0.96
Exercise	5.26	2.70	3.80	0.05
Cool down	5.36	3.23	2.76	0.10
Recovery	9.39	3.64	6.64	0.01
Intensity *×* Phase				
Very Low *×* Recovery	−13.50	5.61	5.79	0.016
Very Low *×* Cool down	−7.90	4.97	2.52	0.11
Very Low *×* Exercise	−7.83	4.15	3.55	0.059
Very Low *×* Warmup	−2.11	3.01	0.49	0.48
Very Low *×* Baseline	Reference			
Low *×* Recovery	−5.65	4.62	1.50	0.22
Low *×* Cool down	−1.09	4.09	0.07	0.79
Low *×* Exercise	−2.00	3.42	0.34	0.56
Low *×* Warmup	0.16	2.47	0.004	0.95
Low *×* Baseline	Reference			
Low/Mod *×* Recovery	−1.79	5.15	0.12	0.73
Low/Mod *×* Cool down	2.64	4.57	0.33	0.56
Low/Mod *×* Exercise	0.14	3.82	0.001	0.97
Low/Mod *×* Warmup	0.93	2.76	0.11	0.74
Low/Mod *×* Baseline	Reference			
Weekly frequency of exercise	3.88	1.80	6.90	0.009

Results were obtained from a generalized estimating equation (GEE), using a model-based estimator structured as an AR1 correlation matrix with a normal probability distribution and an identity link function. * Cardiac force meter (CFM) ranges: Moderate, <37.5; Low/Mod (erate), 37.6–42.5; Low, 42.6–47.5; Very Low, >47.5. ^^^ GEE results are for change from moderate to very low. ^#^ GEE results are for change from baseline to recovery.

## Data Availability

Datasets related to this article can be requested from the corresponding author.

## Supplementary Materials

The following are available online at https://www.mdpi.com/article/10.3390/jpm12040510/s1, Table S1: Mean changes in HEG from phase to phase of exercise session in male; Table S2: Mean changes in HEG from phase to phase of exercise session in female.
