# Supplementary material for: A Novel Intensity-Based Approach to Increasing Prefrontal Cerebral Oxygenation by Walking Exercise
Source: J Pers Med. 2022 Mar 22;12(4):510. doi: 10.3390/jpm12040510 (PMC9027192; doi:10.3390/jpm12040510)
Supplement: Supplementary file 1 [file jpm-12-00510-s001.zip › jpm-1612815-supplementary.pdf]

Journal of Personalized Medicine

MDPI

Table S1. Mean changes in HEG from phase to phase of exercise session in **male** (N = 55).

Variables	β	SE	χ^2	p
Intensity* ^				
Moderate	*Reference*			
Low/Mod	-10.66	9.74	0.62	.27
Low	1.04	8.81	0.01	.91
Very Low	8.45	10.73	1.20	.43
Phase#				
Baseline	*Reference*			
Warmup	12.17	6.02	4.09	.88
Exercise	2.65	5.35	0.24	.46
Cool down	3.34	4.49	0.55	.62
Recovery	-0.51	3.27	0.03	.04
Intensity × Phase				
Very Low × Recovery	-21.08	9.20	5.26	**.02**
Very Low × Cool down	-8.46	8.18	1.07	.30
Very Low × Exercise	-10.32	6.86	2.26	.13
Very Low × Warmup	-5.62	4.99	1.27	.26
Very Low × Baseline	*Reference*			
Low × Recovery	-7.32	7.55	0.94	.33
Low × Cool down	-0.87	6.72	0.02	.90
Low × Exercise	-1.93	5.63	0.12	.73
Low × Warmup	-0.32	4.09	0.006	.94
Low × Baseline	*Reference*			
Low/Mod × Recovery	-10.79	8.35	1.67	.20
Low/Mod × Cool down	-0.11	7.42	<0.001	.99
Low/Mod × Exercise	-0.78	6.23	0.02	.90
Low/Mod × Warmup	0.79	4.53	0.03	.86
Low/Mod × Baseline	*Reference*			
Weekly frequency of exercise	4.20	2.07	4.12	**.04**

Results were obtained from a generalized estimating equation (GEE), using a model-based estimator structured as an AR1 correlation matrix with a normal probability distribution and an identity link function.

*Cardiac force meter (CFM) ranges: Moderate, <37.5; Low/Mod(erate), 37.6–42.5; Low, 42.6–47.5; Very Low, > 47.5.

^ GEE results are for change from moderate to very low.

GEE results are for change from baseline to recovery.

Table S2. Mean changes in HEG from phase to phase of exercise session in **female** (N = 59).

**Variables**	*β*	*SE*	χ^2	*p*
Intensity* ^				
Moderate	*Reference*			
Low/Mod	4.65	8.20	0.32	.57
Low	4.48	7.25	0.38	.54
Very Low	6.78	8.79	0.59	.44
Phase#				
Baseline	*Reference*			
Warmup	0.27	2.06	0.02	.90
Exercise	6.90	2.86	5.83	.02
Cool down	7.69	3.45	4.98	.03
Recovery	7.01	3.91	3.21	.07
Intensity × Phase				
Very Low × Recovery	-6.08	6.06	1.26	.26
Very Low × Cool down	-7.27	5.34	1.85	.17
Very Low × Exercise	-5.50	4.43	1.54	.22
Very Low × Warmup	1.08	3.19	0.11	.74
Very Low × Baseline	*Reference*			
Low × Recovery	-4.32	5.00	0.75	.39
Low × Cool down	-1.03	4.41	0.06	.82
Low × Exercise	-1.87	3.66	0.26	.61
Low × Warmup	0.66	2.63	0.06	.80
Low × Baseline	*Reference*			
Low/Mod × Recovery	6.81	5.64	1.46	.23
Low/Mod × Cool down	5.78	4.97	1.35	.25
Low/Mod × Exercise	1.34	4.12	0.11	.75
Low/Mod × Warmup	1.14	2.96	0.5	.70
Low/Mod × Baseline	*Reference*			
Weekly frequency of exercise	1.86	2.14	0.75	.39

Results were obtained from a generalized estimating equation (GEE), using a model-based estimator structured as an AR1 correlation matrix with a normal probability distribution and an identity link function.

*Cardiac force meter (CFM) ranges: Moderate, <37.5; Low/Mod(erate), 37.6–42.5; Low, 42.6–47.5; Very Low, > 47.5.

^ GEE results are for change from moderate to very low.

GEE results are for change from baseline to recovery.
